# Supplementary material for: The interactome of intact mitochondria by cross-linking mass spectrometry provides evidence for coexisting respiratory supercomplexes
Source: Mol Cell Proteomics. 2017 Dec 8;17(2):216–32. doi: 10.1074/mcp.RA117.000470 (PMC5795388; doi:10.1074/mcp.RA117.000470)
Supplement: Supplemental Data [file supp_RA117.000470_133922_0_supp_23978_fzffwf.zip › spectra_annotation/mito_DR_spectra_annotation/68-1-6-1-6-1.pdf]

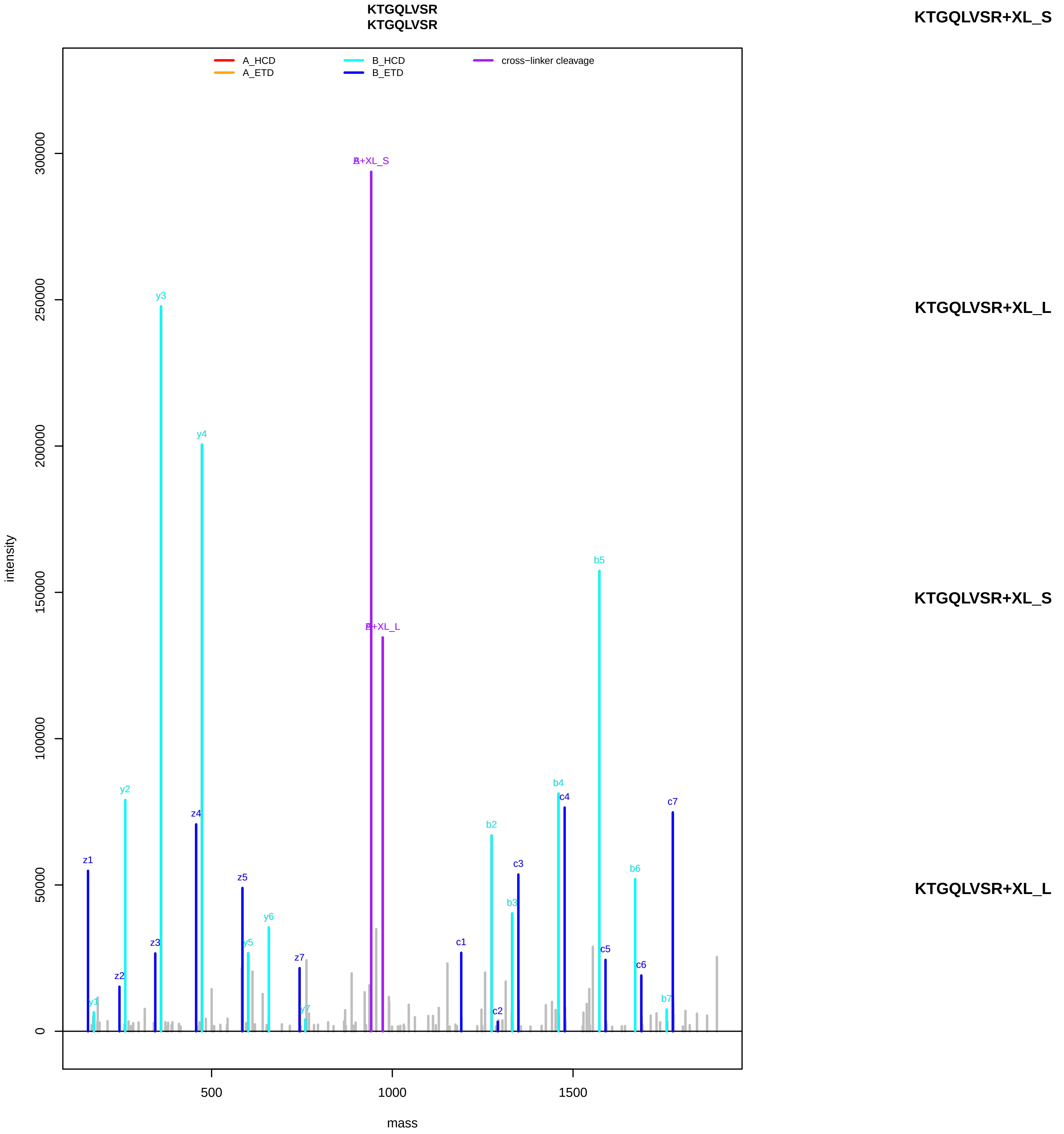
KTGQLVSR
KTGQLVSR
A_HCD
A_ETD
B_HCD
B_ETD
cross−linker cleavage
intensity
mass
0
50000
100000
150000
200000
250000
300000
500
1000
1500
z1
y1
y2
z2
z3
y3
z4
y4
z5
y5
y6
z7
y7
B+XL_S
B+XL_L
c1
b2
c2
b3
c3
b4
c4
b5
c5
b6
c6
b7
c7
KTGQLVSR+XL_S
KTGQLVSR+XL_L
KTGQLVSR+XL_S
KTGQLVSR+XL_L
